# In Vitro Antiviral Activity of Red Algae Extracts from *Chondracanthus teedei* var. *lusitanicus* and *Osmundea pinnatifida* Against Coxsackievirus A12 and a Lentiviral Vector

**DOI:** 10.3390/tropicalmed11020041

**Published:** 2026-01-31

**Authors:** Nanci Santos-Ferreira, Clévio Nóbrega, Marta Mota, Luís Pereira de Almeida, Leonel Pereira, Teresa Gonçalves, Célia Nogueira

**Affiliations:** 1 Institute of Microbiology, Faculty of Medicine, University of Coimbra, 3004-504 Coimbra, Portugal; 2 CNC—Centre for Neuroscience and Cell Biology, University of Coimbra, 3004-504 Coimbra, Portugal; 3 Virus-Host Interactions & Therapeutic Approaches (VITA) Research Group, KU Leuven-Department of Microbiology, Immunology and Transplantation, Rega Institute, 3000 Leuven, Belgium; 4 Algarve Biomedical Center Research Institute (ABC-RI), University of Algarve, 8005-139 Faro, Portugal; 5 Faculty of Medicine and Biomedical Sciences, University of Algarve, 8005-139 Faro, Portugal; 6 Faculty of Pharmacy, University of Coimbra, 3000-548 Coimbra, Portugal; 7 Marine Resources, Conservation and Technology, Marine Algae Lab., Centre for Functional Ecology-Science for People & the Planet (CFE), Department of Life Sciences, University of Coimbra, 3000-456 Coimbra, Portugal; leonel.pereira@uc.pt

**Keywords:** antiviral activity, algae, *Chondracanthus teedei* var. *lusitanicus*, *Osmundea pinnatifida*, Coxsackievirus A12, lentivirus

## Abstract

Infectious diseases remain a major global health challenge, underscoring the need for safe and accessible antiviral therapies. Natural products, particularly marine macroalgae, are promising sources of bioactive compounds with antiviral properties. This study evaluated the antiviral activity of extracts from two red algae collected along the Portuguese coast: two life stages (tetrasporophyte and female gametophyte) of *Chondracanthus teedei* var. *lusitanicus* and the algae *Osmundea pinnatifida*. Antiviral effects were assessed against Coxsackievirus A12 (CVA12) and a lentivirus (LV) vector model. Extracts from both algae inhibited viral replication in vitro at non-cytotoxic concentrations. The tetrasporophyte extract of *C. teedei* exhibited virucidal activity against CVA12, and the results are consistent with interference with multiple stages of the viral life cycle, while also inducing an antiviral state in HEK-293T cells against LV infection. The female gametophyte extract affected early stages of CVA12 and LV infection and showed potential virucidal activity. *O. pinnatifida* demonstrated the strongest antiviral effects against both viruses. These findings highlight the antiviral potential of these red algal extracts and warrant further in vivo evaluation.

## 1. Introduction

Over recent decades, remarkable advances in medicine have improved the management and prevention of numerous diseases. Nevertheless, infectious diseases continue to pose a major global health threat, particularly due to the emergence of drug-resistant pathogens and unequal access to treatment in low- and middle-income countries.

Since the approval of the first antiviral agent, idoxuridine, in 1963, approximately one hundred distinct antiviral compounds have been developed and are currently available for the treatment of various viral infections [1]. However, the continuous emergence and re-emergence of viral outbreaks, many of them life-threatening, such as human immunodeficiency virus (HIV), Ebola, Zika, influenza, Middle East respiratory syndrome coronaviruses (MERS-CoV), and most notably the COVID-19 pandemic caused by severe acute respiratory syndrome 2 (SARS-CoV-2), have underscored the urgent need for novel antiviral agents. These outbreaks have not only highlighted the limitations of current antiviral drugs but also exposed vulnerabilities in global preparedness. The growing resistance to existing antivirals observed in viruses such as HIV, influenza, and herpes simplex virus (HSV) [2] reinforce the necessity to explore new, safe, and accessible therapeutic options.

In this context, natural products have gained significant attention for drug discovery due to their chemical diversity, broad biological activity, and historical use in traditional medicine. In particular, marine ecosystems, including seaweeds, offer a vast and largely untapped reservoir of bioactive compounds with demonstrated antibacterial, antifungal, antiviral, antiparasitic, anticancer, and anti-inflammatory properties [3,4]. Marine macroalgae, or seaweeds, are especially promising due to their production of sulphated polysaccharides and secondary metabolites with potent antiviral effects [5,6].

Several studies have demonstrated the antiviral potential of seaweed-derived compounds against a wide range of viruses, including HSV-1 and HSV-2 [7,8], Newcastle disease virus [9], human metapneumovirus (HMPV) [10], HIV [8,11], hepatitis C virus (HCV) [12], human papillomavirus (HPV) [11], dengue virus [13], and even coronaviruses such as SARS-CoV and MERS-CoV [11]. These compounds often act through virus entry inhibition, replication interference, or immune modulation. The earliest reports of antiviral activity from seaweed-derived polysaccharides date back more than 60 years, with Gerber et al. [14] demonstrating protection against mumps and influenza B viruses.

More recently, the COVID-19 pandemic has reignited global interest in marine-derived antivirals. Several studies have explored seaweed polysaccharides, phlorotannins, and lectins for their ability to inhibit SARS-CoV-2 spike protein interaction with ACE2 receptors, viral replication, and inflammatory responses [15,16,17]. These findings further emphasize the potential of marine natural products as a frontline strategy in preparing for future pandemics.

Coxsackievirus A12 (CVA12) is a non-enveloped, positive-sense, single-stranded RNA virus classified within the genus *Enterovirus* of the family *Picornaviridae* [18]. Although infections caused by CVA12 are reported less frequently than those attributed to other enteroviruses, this serotype has been implicated in cases of hand, foot, and mouth disease, herpangina [19], and acute flaccid paralysis [20].

Human immunodeficiency virus, classified within the genus Lentivirus, remains a major global health threat, responsible for over 40 million deaths worldwide and persisting as a widespread epidemic despite significant medical advances [21]. Lentiviral vectors provide a safer, controllable model for antiviral evaluation, enabling HIV-related studies [22].

Among marine algae, red seaweeds (Rhodophyta) are known to produce high levels of bioactive compounds such as carrageenans, floridosides, and bromophenols, which exhibit antiviral activity with low cytotoxicity, high biocompatibility, and biodegradability—making them attractive candidates for therapeutic development [2,16,23]. Moreover, the low production costs and broad availability of these natural resources could facilitate equitable access to treatment, particularly in resource-limited settings.

In this study, we evaluated the antiviral activity of three extracts from two algae collected along the Portuguese coast: two life stages—tetrasporophyte and female gametophyte—of the red alga *Chondracanthus teedei* var. *lusitanicus* (*C. teedei* var. *lusitanicus)* (order Gigartinales) and the red alga *Osmundea pinnatifida* (*O. pinnatifida*) (order Ceramiales). The antiviral activity of these extracts was tested against CVA12 and a lentivirus vector (LV) model to simulate retroviral infection.

## 2. Materials and Methods

### 2.1. Natural Extracts

The *C. teedei* var. *lusitanicus* extracts were prepared as previously described by Soares et al. [24]. Samples of this species were washed on site to remove sand and salt, placed in plastic bags, and transported in a cooler. In the laboratory, all material was rinsed with autoclaved distilled water and gently cleaned with a cotton swab to eliminate residual salts, epiphytes, and debris from the thallus surface. Thalli were then sorted into life-cycle phases, tetrasporophytes, fructified female gametophytes, and non-fructified thalli, using a magnifying glass. Tetrasporophytes were identified by the presence of tetrasporangial sori, visible as dark red spots on the thallus surface, main axis, and lateral branches. Fructified female gametophytes displayed prominent spherical cystocarps producing carpo-spores, whereas non-fructified thalli lacked cystocarps. Only thalli exceeding 5 cm in length were selected to minimize misidentification among phases [24].

For alkali extraction, ground dry material was weighed on a Kern scale, and 1 g sam-ples of both tetrasporophyte and female gametophyte phases were used (*n* = 3). Prior to extraction, each 1 g portion was rehydrated and pretreated with a 1:1 mixture of acetone and methanol (75 mL each) at room temperature for 12 h to remove the organo-soluble fraction. Samples were then transferred to 150 mL of NaOH (1 M) and heated in a water bath at 85–90 °C for 3 h. The hot solutions were vacuum-filtered twice through a cloth fil-ter. Carrageenans were precipitated by adding ethanol (96%) at twice the volume of the warm extract, producing a whitish coagulum that was dried at 50–60 °C for 24 h [24].

The resulting carrageenan extracts (from fructified tetrasporophytes and female gametophytes) were ground with a mortar and pestle. A stock solution of 10 g/L was pre-pared in ultrapure water, aliquoted into 1.5 mL microtubes, and stored at −20 °C until further use. The extract of the female gametophyte stages is characterized by the presence of a hybrid kappa/iota/mu/nu carrageenan, while the extract of tetrasporophyte stages produce a hybrid xi/theta carrageenan [24].

The *O. pinnatifida* n-hexane extract was obtained according to the extraction procedure previously reported by Silva et al. [25] Freeze-dried seaweed samples were ground into a fine powder and subjected to sequential extraction using organic solvents of different polarities, namely methanol (>99%), dichloromethane (>99%), and n-hexane (99%). The powdered material was transferred to a beaker, and methanol was added at a 4:1 solvent-to-sample ratio (mL/g). Extraction proceeded for 12 h at room temperature under continuous stirring and protected from light with aluminum foil. The mixture was then filtered through Whatman™ filter paper (Marlborough, MA, USA), yielding the methanol extract (filtrate) and the residual biomass.

After drying at room temperature, the residue was placed in a clean beaker, dichloromethane was added at the same 4:1 ratio (mL/g), and extraction was carried out under the previously described conditions. Filtration produced the dichloromethane extract. The methanol extract was subsequently transferred to a separatory funnel, and n-hexane was added in a 1:1 (*v*/*v*) proportion to perform a liquid–liquid extraction. This procedure partitioned the methanol extract into two fractions: a methanol fraction and a hexane fraction. All extracts and fractions were evaporated to dryness under reduced pressure using a rotary evaporator (Laborota 4000, Heidolph, Schwabach, Germany) at 40 °C, followed by exposure to a gentle nitrogen stream directed onto the sample surface to ensure complete solvent removal. The dried n-hexane extract was dissolved to a concentration of 6.4 g/L in DMSO [25].

### 2.2. Cells and Viruses

Human Colorectal Adenocarcinoma cells Caco-2 (Caco-2) (ATCC HTB-37) were obtained from American Type Culture Collection (ATCC). Caco-2 cells were grown in Dulbecco’s Modified Eagle’s Medium (DMEM) containing 20% heat-inactivated fetal bovine serum (FBS, Belize City, Belize; Gibco, Waltham, MA, USA), 100 U/mL and 100 µg/mL penicillin-streptomycin (Gibco), 12 mM HEPES (Sigma-Aldrich, St. Louis, MO, USA) and 10 mM of sodium bicarbonate (Sigma-Aldrich).

HEK-293T (ATCC CRL-3216) cells were obtained from ATCC. HEK-293T cells were grown in DMEM containing 10% heat-inactivated FBS, 100 U/mL and 100 µg/mL penicillin-streptomycin and 3.7 g/L sodium bicarbonate.

Human CVA12, strain Texas 12 (ATCC #VR-1018) (10^6.6^ TCID_50_/mL) was obtained from ATCC and propagated in Caco-2 cells with DMEM containing 20% of heat-inactivated FBS and 100 U/mL and 100 µg/mL penicillin-streptomycin at 37 °C in a humidified atmosphere of 5% CO_2_.

The LV used for experiments was a self-inactivating lentiviral transfer vector encoding for the enhanced green fluorescent protein (eGFP). LV was propagated in HEK-293T cells with DMEM containing 10% of heat-inactivated FBS and 100 U/mL and 100 µg/mL penicillin–streptomycin at 37 °C in a humidified atmosphere of 5% CO_2_. LV concentration was determined by p24 antigen ELISA (HIV-ELISA, Zeptometrix, Buffalo, NY, USA) [26].

### 2.3. Cytotoxicity Assays

The cytotoxicity of the algae extracts and their solvents, ultrapure water and DMSO (Supplementary Figure S1) was evaluated on both cell lines using a non-toxic, redox-based assay that measures cell viability (alamarBlue^®^ assay; Invitrogen, Carlsbad, CA, USA). Briefly, cells (100,000 cells/well) were seeded in a 24-well plate (Corning, New York, NY, USA) and incubated for 24 h (HEK-293T) or 36 h (Caco-2) at 37 °C. Different concentrations of the extracts (1, 10, 50, 100 and 200 µg/mL) were added to the cells and incubated for 8, 24 and 48 h. In cases where cytotoxicity was observed at 48 h, the incubation was prolonged up to 72 h. After, the medium was substituted for a solution of 10% resazurin sodium salt (Sigma-Aldrich), incubated for 2 h at 37 °C with 5% CO_2_, and the absorbance of the supernatants was read at wavelengths of 570 and 600 nm (SPECTRAmax PLUS 384, Molecular Devices, San Jose, CA, USA). Cell viability was calculated in relation to the control group which did not receive treatment.

### 2.4. Anti-Coxsackievirus A12 Activity

#### 2.4.1. Time-of-Addition Antiviral Assays

To address the effect of extracts on CVA12 infection, four treatment protocols were employed. Briefly, Caco-2 cells (200,000 cells/well) were seeded into 24-well plates and incubated for 24 h at 37 °C with 5% CO_2_. Extract samples were included in the assay for one of the following times: (i) pre-treatment—extract pre-incubated with cells for 2 h and removed prior to virus; (ii) co-treatment—extract added simultaneously with virus and incubated for 2 h; (iii) post-treatment—extract added 2 h after infection and included for the duration of the assay; and (iv) virucidal treatment—extract pre-incubated with virus for 2 h at 4 °C prior to infection and included during virus adsorption [27]. Infection was performed with 1.2 × 10^5^ CVA12 particles per condition. Extracts’ antiviral activity was assessed at their highest tested non-cytotoxic concentrations. Specifically, the tetrasporophyte and female gametophyte extracts were tested at 200 μg/mL, whereas the *O. pinnatifida* extract was evaluated at 100 μg/mL. Cells and culture supernatants were collected after 48 h post-infection and viral RNA load was determined by RT-qPCR.

#### 2.4.2. Reverse Transcriptase Real-Time PCR Assay

Quantitative real-time polymerase chain reaction (qPCR) was used to determine the CVA12 viral load in infections assays. Viral RNA was extracted from the supernatant using the MagNA Pure Compact Nucleic Acid Isolation Kit (Roche, Basel, Switzerland) on the automatic extractor MagNA Pure Compact System (Roche). Reverse transcription reaction was performed using random hexamer primer with the Transcriptor First Strand cDNA Synthesis kit (Roche) according to the manufacturer’s instructions. The resulting cDNAs were used as templates for qPCR, which was carried out on LightCycler 2.0 instrument (Roche) using the FastStart DNA Master SYBR Green I kit (Roche). CVA12 expression was calculated relative to 18S mRNA expression, using primers described elsewhere [28] and applying the 2^−ΔΔCt^ formula [29]. A control with only cells and cells infected with CVA12 without treatment was included.

### 2.5. Anti-Lentivirus Activity

#### 2.5.1. Time-of-Addition Assays

To address the effect of extracts on a genetically modified LV, three treatment protocols were employed. Briefly HEK-293T cells (100,000 cells/well) were seeded in 12-well plates (Corning) with coverglass coated with Poly-L-Lysine (Gibco) and incubated for 24 h at 37 °C with 5% CO_2_. Since the lentivirus used was genetically modified to not replicate after cell entry, only three conditions were tested: (i) pre-treatment—the extracts were pre-incubated with cells for 2 h and removed prior to infection with LV; (ii) co-treatment—extracts were added to the cells simultaneously with the virus and removed 12 h after infection; and (iii) virucidal treatment—the extracts were pre-incubated with virus for 2 h at 4 °C prior to infection [27]. Infection was performed with 200 ng LV per condition. The rate of infection (infected cells/total cells) was evaluated by fluorescence microscopy.

#### 2.5.2. Immunofluorescence Assay

HEK-293T cell infection by LV was quantified using fluorescence microscopy [30]. After 72 h post-infection, HEK-293T cells were washed twice with PBS and incubated with 4% formaldehyde (Sigma-Aldrich) for 15 min at room temperature. After, cells were washed with PBS and incubated with 4′,6-diamidino-2-phenylindole (DAPI) for 15 min, protected from light. Cells were washed again with PBS and the cover-glass was assembled on microscope slides with DACO mounting medium (Palex Medical SA, Madrid, Spain). After this, slides were sealed with polish and observed on an Axio Imager Z2 (Zeiss, Oberkochen, Germany). A control with only cells and LV-infected cells without treatment was included. Ten pictures of each cover-glass were taken covering the cover-glass on cross with the objective Zeiss Plan-Apochromat 20×, in brightfield, DAPI and EGFP channels. Total cell numbers (DAPI positive) and infected cells (GFP positive) were counted using Zeiss software (ZEN 3.13).

### 2.6. Statistical Analysis

Numerical results are reported as mean + SD. Statistical analysis was performed using GraphPad Prism 10.2.0 (GraphPad Software, Inc., San Diego, CA, USA). The specific methods of statistical analysis and *p* values are indicated in the figure legends or the text. For cell viability assays, half maximal cytotoxic concentration (CC_50_) was calculated by non-linear regression (curve fit) with GraphPad using the dose–response curves from the experimental data, expressed with 95% confidence intervals.

## 3. Results and Discussion

### 3.1. Cytotoxicity Assays

**Figure 1:**
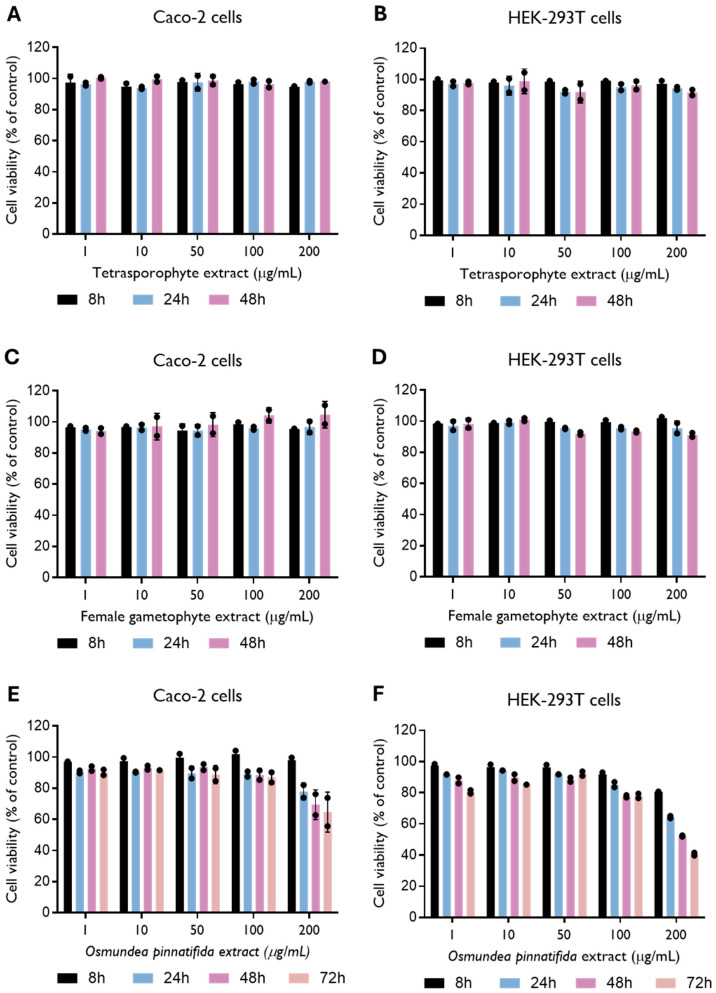
Effect of tetrasporophyte (**A**,**B**), female gametophyte (**C**,**D**) and *Osmundea pinnatifida* (**E**,**F**) extracts on Caco-2 and HEK-293T cell viability up to 48 and 72 h of incubation. Data were obtained by resazurin metabolization and are presented as mean ± SD from two independent experiments (*n* = 2), with each carried out in quadruplicate.

To evaluate extract toxicity, Caco-2 cells and HEK-293T cells were treated with several concentrations of the natural extracts. The tetrasporophyte and female gametophyte extracts of the alga *C. teedei* var. *lusitanicus* showed no toxicity in Caco-2 and HEK-293T cells (CC_50_ > 200 µg/mL) up to 48 h of incubation (Figure 1A–D). In the Gigartinales order, to which *C. teedei* var. *lusitanicus* belongs, the tetrasporophytic thalli synthesize carrageenans of the lambda family, whereas gametophytic thalli are characterized by the production of kappa/iota hybrid carrageenans [31]. The content of *C. teedei* comprises approximately 30% of kappa/iota carrageenans and about 58% of xi/theta carrageenans [32]. No evidence of toxicity has been reported for various carrageenan types, whether isolated from distinct algal species and life stages [7,33,34]. A recent review by Lee of in vitro and in vivo safety data reports half-maximal cytotoxicity concentrations (CC_50_) ranging from 5 to 3000 μg/mL [35]. Our results corroborate these findings, as no cytotoxic effects were detected at the highest concentration tested (200 μg/mL). On the other hand, the *O. pinnatifida* extract showed toxicity in both cell lines (Figure 1E,F), resulting in a CC_50_ of 369.0 (235.6–14,802) µg/mL and 170.7 (128.1–442.2) µg/mL in Caco-2 and HEK-293T cells, respectively, at 48 and 72 h (Supplementary Figure S2). Alterations in HEK-293T cell morphology, i.e., inhibition of cell cytoplasmic extensions, were detected when cells were incubated with *O. pinnatifida* extract at a concentration of 200 μg/mL (Supplementary Figure S3). Thus, further experiments with *O. pinnatifida* extract were performed at a concentration of 100 µg/mL. Consistent findings were reported by Barreto et al., who observed cytotoxic effects of the hexane fraction of *O. pinnatifida*, with CC_50_ values lower than 200 µg/mL against HeLa cells [36].

### 3.2. Antiviral Activity Against Coxsackievirus A12

**Figure 2:**
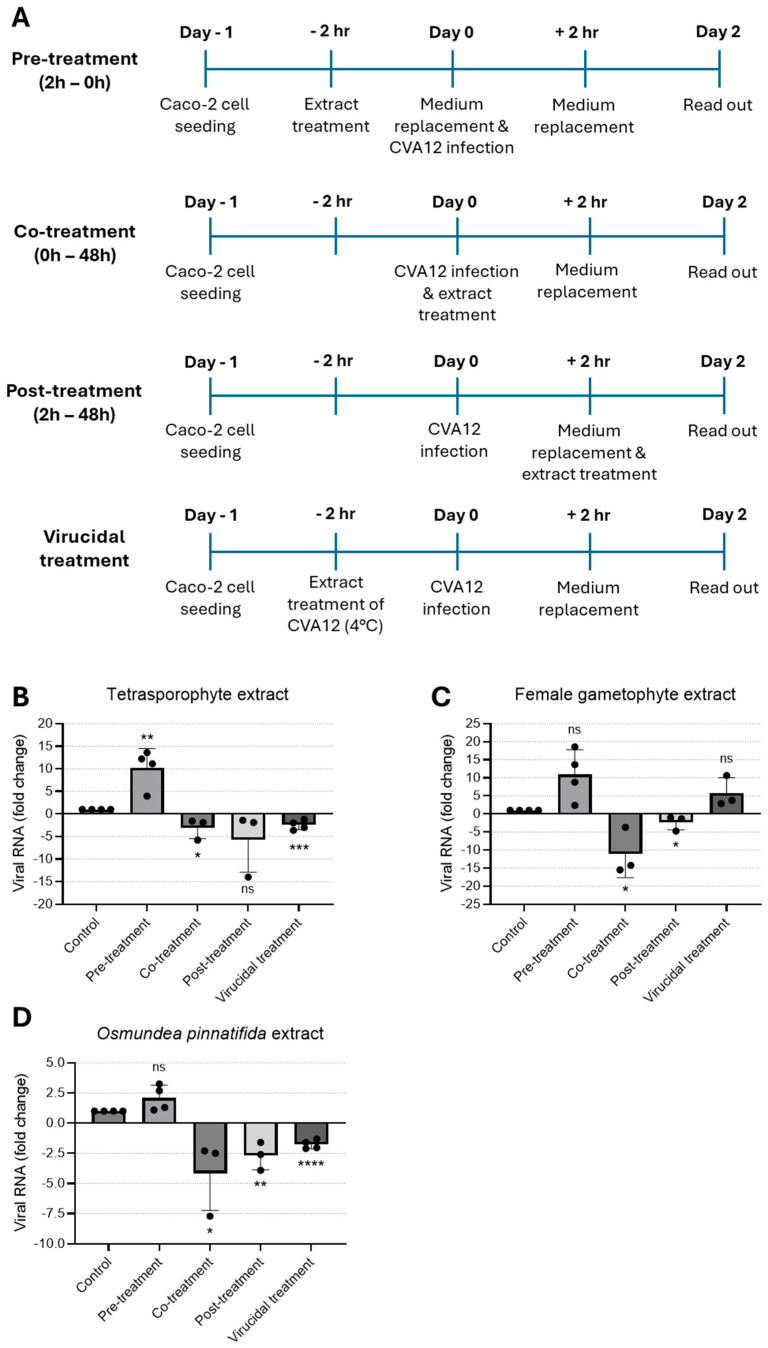
Time-of-addition assay to evaluate the antiviral effect of natural extracts against CVA12. (**A**) Schematic representation of time-of-addition assays. (**B**–**D**) Antiviral activity of (**B**) tetrasporophyte 200 µg/mL, (**C**) female gametophyte 200 µg/mL, and (**D**) *O. pinnatifida* 100 µg/mL extracts against CVA12 infection in Caco-2 cells determined by RT-qPCR. Results are expressed as fold change in viral RNA load due to treatment. Data are presented as mean + SD from at least three independent experiments (*n* ≥ 3) each carried out in triplicate. Data were analyzed using an unpaired Student’s *t*-test for comparison of antiviral activity between treatment and control. Given the small sample size, normality could not be formally assessed; therefore, results should be interpreted with caution. ns, not significant; *, *p* < 0.05; **, *p* < 0.01; ***, *p* < 0.001; and ****, *p* < 0.0001.

To examine the impact of the tetrasporophyte and female gametophyte extracts of the red alga *C. teedei* var. *lusitanicus*, and n-hexane extract of *O. pinnatifida* alga on virus replication, CVA12-infected Caco-2 cells were treated with extracts at the maximum non-toxic concentration tested at different time-points. In an effort to identify the stage of the CVA12 life cycle that is affected by the treatment of extracts, we designed pre-treatment (extracts added to cells for a period of 2 h prior to infection), co-treatment (extracts added together with virus for 2 h), post-treatment experiments (extracts added 2 h after virus infection), and virucidal treatment (extracts incubated with virus for 2 h prior to infection, Figure 2A). Cells and culture supernatants were harvested for 2 days post-infection and viral RNA levels were evaluated by qRT-PCR. As shown in Figure 2B, pre-treatment of cells with the tetrasporophyte extract prior to CVA12 infection did not confer cell protection; instead, viral replication was significantly increased. In contrast, the addition of the tetrasporophyte extract during infection (co-treatment) resulted in a significant reduction in viral RNA load, whereas post-treatment also greatly decreased viral RNA load, albeit not significantly. Moreover, incubation of the extract with the virus prior to infection resulted in a 2.5-fold reduction (*p* < 0.0001) in viral RNA load. These data suggested that the tetrasporophyte extract exhibits a significant virucidal activity and may also interfere in the early stages of virus replication, possibly by inhibiting the attachment of virions to the cell surface or by having an inhibitory effect on post-attachment, either by blocking the virion regions necessary for interaction with host–cell receptors or by impairing the conformational transitions that the viral particle must undergo to initiate infection [37,38]. The marked reduction in viral RNA load observed in the post-infection assay suggests that the extract may also exert an inhibitory effect on viral synthesis. Considering the replication kinetics of other enteroviruses, such as Enterovirus 71 (EV-71) [39] and CVA16 [40], that indicate that these enteroviruses first attach, uncoat, and enter the host cell following the 1 h adsorption period, by 3 h post-infection, the levels of total intracellular viral RNA are already constitutively increased. So, this inhibitory activity could be attributed to the suppression of viral protein synthesis essential for virus replication or the inhibition of viral RNA synthesis [33].

When the female gametophyte extract was added to the cell culture before virus infection (pre-treatment) or pre-incubated with the virus (virucidal treatment), no protection against CVA12 infection was observed; conversely, a marked enhancement in viral RNA load was observed relative to the control condition (Figure 2C). On the other hand, addition of the female gametophyte extract during virus infection (co-treatment) and 2 h after infection (post-treatment) resulted in a 10- and 2.4-fold reduction in viral RNA load, respectively. These data suggested that the female gametophyte extract could act in the early stages of virus infection, possibly due to the ability to block the internalization of the nucleocapsid into the host cell cytoplasm, likely because virions that enter the cells are unable to undergo uncoating and subsequent release from endosomes [41]. Similarly to the effects observed with the tetrasporophyte extract, the reduction in viral RNA load during post-treatment assays suggests that the female gametophyte extract exerts an inhibitory effect on viral synthesis.

Treatment with *O. pinnatifida* extract led to a marked decrease in viral RNA load when added during infection (co-treatment) and 2 h after infection (post-treatment), yielding 4.1- and 2.7-fold reduction in viral RNA load, respectively (Figure 2D). When the extract was added to the cell culture before virus infection (pre-treatment), no protection against CVA12 was observed. By contrast, incubation of the extract with the virus prior to infection resulted in a 1.8-fold reduction in viral RNA load. These data suggested that the *O. pinnatifida* extract displays an activity profile comparable to that of the tetrasporophyte extract, demonstrating a significant virucidal activity and potential interference with early events of the viral replication cycle. This interference may occur through inhibition of virion attachment to the host cell surface or through post-attachment blockade, either by masking regions of the viral particle required for receptor engagement or by disrupting the conformational rearrangements essential for initiating infection [37,38]. Moreover, the significant reduction in viral RNA load detected in the post-infection assay indicates that the extract may also impair subsequent steps of viral synthesis.

Red algal polysaccharides comprise various sulfated galactans, sulfated rhamnans or mannans, carrageenans, and agars [8]. Among these, agar and carrageenan constitute the major matrix polysaccharides [8,42]. Carrageenans have been reported to have a broad antiviral spectrum including pathogenic viruses [33]. Notably, some reports have shown that sulfated polysaccharides can, unexpectedly, potentiate viral infection, thereby posing a substantial constraint to their therapeutic development [43,44,45]. These observations are derived from both in vitro and in vivo studies using a HIV model, and appear to be a dose-dependent effect. Nevertheless, the precise molecular mechanisms underlying the concentration-dependent pro- and antiviral activities of these compounds remain to be elucidated. In the CVA12/Caco-2 cell model, pre-treatment of the cells with tetrasporophyte and female gametophyte extracts resulted in a comparable outcome, as we observed an enhancement of the viral RNA load. This effect was likewise evident for the female gametophyte extract under the virucidal treatment condition. Furthermore, in a study conducted with two other red algal species, pre-treatment of Vero cells with the extracts prior to herpes simplex virus type 1 (HSV-1) infection failed to confer any detectable cellular protection [8].

Carrageenans exhibit selective antiviral properties against both enveloped and non-enveloped viruses, mainly by disruption of viral attachment and subsequent internalization processes, thereby preventing the entry of viral particles into host cells [28,32]. In this study, we report the virucidal activity of tetrasporophyte and *O. pinnatifida* extracts, although it has been documented that most algae sulfated polysaccharides do not exhibit a significant capacity to directly inactivate virions [32,46,47]. It is believed that the virucidal activity of sulphated polysaccharides is caused by the formation of a stable virion-sulphated polysaccharide complex, in which the sites on the viral envelope necessary for the virus to bind to host cells are occupied by the sulphated polysaccharide [7,48]. For non-enveloped viruses, an analogous mechanism has been proposed, which consists of blocking the interaction between the viral capsid and cell surface receptors, due to the obstruction of capsid regions involved in binding to cell receptors [37,38]. In the study conducted by Chiu and co-authors, kappa-carrageenan was shown to exhibit strong inhibitory activity against another enterovirus, EV-71. The authors proposed that this antiviral effect may be mediated through the formation of virus–polysaccharide complexes, which impede viral entry into host cells [33]. Similarly, a polysaccharide extracted from a green alga can also inactivate EV-71 by binding to virus particles and blocking some early steps of the virus life cycle [49]. In addition, recent in vitro studies demonstrated that carrageenans strongly inhibit the entry of SARS-CoV-2 into cells, primarily by disrupting the interaction between the viral spike protein and the host cell ACE2 receptor [17,50,51]. When Caco-2 cells were co-exposed to CVA12 and the extracts for 2 h, antiviral activity could be assessed at the stages of viral adsorption and internalization. Under these conditions, all extracts demonstrated robust antiviral effects. These findings may be attributed to concurrent mechanisms, including interference with viral receptor engagement by binding and blocking viral receptors and inhibition of the uncoating process. Evidence from previous studies supports the involvement of these early-stage events: Talarico and Damonte reported that λ-carrageenans impede dengue virus type 2 internalization, presumably because incoming virions are unable to undergo uncoating and escape from endosomes [41]. Similarly, Luo et al. showed that λ-carrageenan suppresses conformational rearrangements of the rabies virus glycoprotein, thereby preventing glycoprotein-mediated cell fusion and hindering subsequent viral uncoating [52]. Bouhlal and colleagues investigated the antiviral properties of two red algal species belonging to the Gigartinales and Ceramiales orders against HSV-1 [8]. Their observations are parallel to our findings, as the simultaneous addition of the polysaccharides and the viral inoculum resulted in a marked protective effect on Vero cells [8].

The three extracts exhibited marked antiviral efficacy when added 2 h after CVA-12 infection, indicating a potential inhibitory effect on viral synthesis. This observation is consistent with previous reports describing antiviral activity at this stage of the replication cycle. For example, Chiu and co-workers demonstrated that k-carrageenans suppress EV-71 mRNA synthesis in Vero cells [33], while Wang and colleagues reported that a polysaccharide derived from green algae significantly decreased EV-71 RNA expression at multiple time points post-infection [49]. Similarly, Pliego-Cortés et al. showed that an extract from the red alga *Halymenia floresii* conferred moderate antiviral activity in an HSV-1 infection model [53] and Wang and co-authors further documented that carrageenans inhibit influenza A virus mRNA and protein synthesis following viral internalization [54].

### 3.3. Antiviral Activity Against Lentivirus

**Figure 3:**
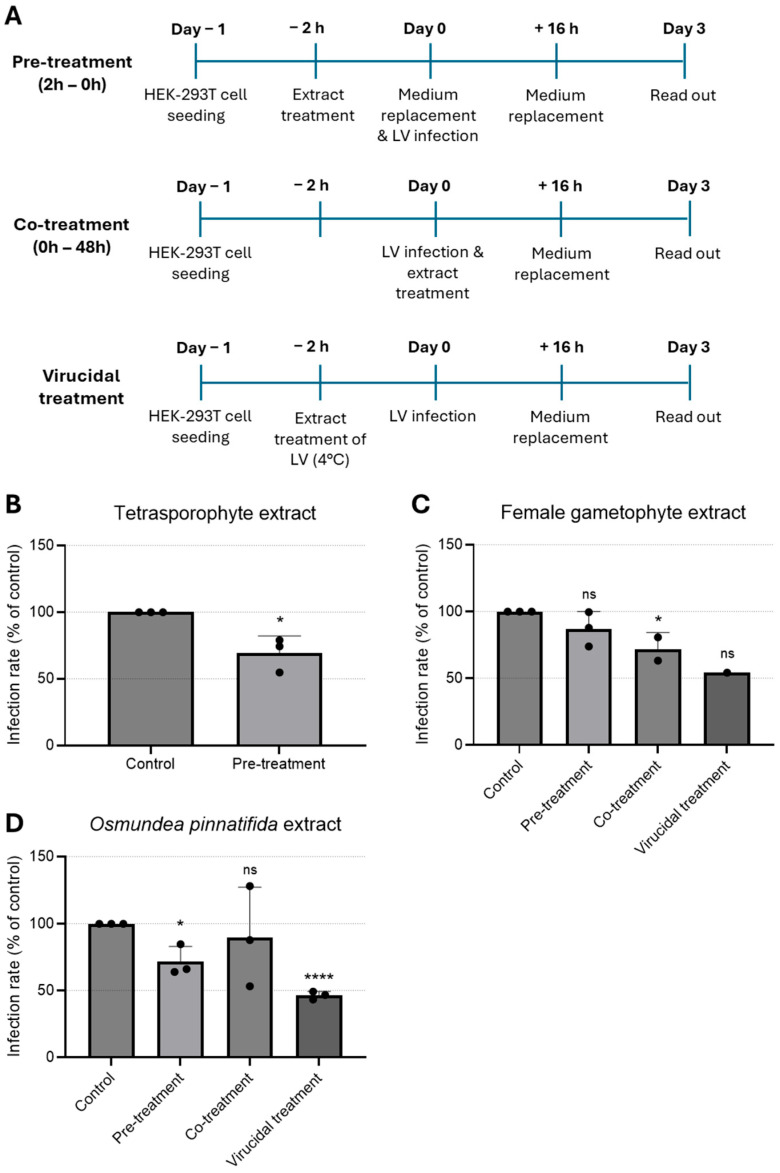
Time-of-addition assay to evaluate antiviral effect of natural extracts against lentivirus. (**A**) Schematic representation of time-of-addition assays. (**B**–**D**) Antiviral activity of (**B**) tetrasporophyte 200 µg/mL, (**C**) female gametophyte 200 µg/mL, and (**D**) *O. pinnatifida* 100 µg/mL extracts against GFP-tagged lentivirus infection in HEK-293T cells determined by immunofluorescence. Results are expressed as infection rate. Data are presented as mean + SD from at least three independent experiments (*n* ≥ 3), with each carried out in triplicate. Data were analyzed using an unpaired Student’s *t*-test (GraphPad Prism 6) for comparison of antiviral activity between treatment and control. Given the small sample size, normality could not be formally assessed; therefore, results should be interpreted with caution. ns, not significant; *, *p* < 0.05; and ****, *p* < 0.0001. LV—lentivirus.

To examine the impact of the extracts on a genetically modified LV model, infected HEK-293T cells were treated with the extracts at the maximum non-toxic concentration tested at different time-points. Since the LV used was self-inactivating, extracts were tested as pre-treatment (extracts added to cells for a period of 2 h prior to infection), co-treatment (extracts added together with virus for 16 h), and virucidal treatment (extracts incubated with virus for 2 h prior to infection, Figure 3A). Infection rate was determined by quantification of infected cells, i.e., cells positive for eGFP. Pre-treatment of cells with the tetrasporophyte extract resulted in a reduction in the (~25) % of infected cells (Figure 3B), hinting that this extract could induce an antiviral state in HEK-293T cells. As shown in Figure 3C, cells pre-treated with the female gametophyte extract prior to lentivirus infection did not confer cell protection. In contrast, addition of the female gametophyte extract during infection (co-treatment) resulted in a significant reduction in the infected cells. Likewise, the female gametophyte extract exhibited pronounced virucidal activity, resulting in an approximate 50% reduction in the proportion of infected cells.

Treatment with *O. pinnatifida* extract resulted in a significant reduction in infection rates, both when cells were pre-incubated with the extract (pre-treatment) and, more prominently, when the extract was pre-incubated with the virus (virucidal treatment) (Figure 3D). These data suggested that the *O. pinnatifida* extract may induce an antiviral state in the cells and that can also block virus infectivity.

Our data on the antiviral activity of the extracts tested against LV indicate a marked virucidal effect, accompanied by the induction of a cellular antiviral state, albeit to a lesser extent. One possible interpretation of these findings is that the extracts may exert a direct inhibitory action on the virion itself, or alternatively, a combined effect involving both the viral particle and cellular receptor sites [7,32]. In the case of sulphated polysaccharides, their antiviral activity is thought to arise from specific interactions with positively charged domains on viral glycoproteins. Such interactions effectively occlude these domains, thereby preventing the virus from engaging with negatively charged cellular receptors [8,47,55]. Concurrently, sulphated polysaccharides extracted from Gigartinales algae have been demonstrated to act as potent and selective inhibitors of HIV-1 replication in cell culture systems [8,56,57].

A limitation of this study is that no phytochemical analyses were performed on the tested extracts, and the work relies on previously published characterizations. This restricts the interpretation of the observed antiviral effects and should be considered when evaluating the findings.

## 4. Conclusions

Seaweed-derived extracts offer several advantages as antiviral agents, including broad natural availability and favorable biopharmaceutical characteristics, such as biodegradability, biocompatibility, safety, and lack of cytotoxicity. Extracts obtained from *C. teedei* var. *lusitanicus* and *O. pinnatifida* inhibited the replication of CVA12 and a modified lentivirus in vitro at concentrations that did not compromise cell viability. The tetrasporophyte extract of *C. teedei* var. *lusitanicus* displayed virucidal activity against CVA12 and was capable of interfering with multiple steps of the CVA12 life cycle. Moreover, it induced an antiviral state in HEK-293T/LV infection. The female gametophyte extract of *C. teedei* var. *lusitanicus* exhibited antiviral effects during the early steps of CVA12 infection and may also impair subsequent viral RNA and protein synthesis. In addition, it inhibited the initial steps of the LV replication cycle and demonstrated a potential virucidal effect. Among the extracts tested, *O. pinnatifida* showed the most pronounced antiviral activity, effectively inhibiting both viruses under different experimental conditions.

Overall, these findings provide preliminary evidence supporting the antiviral potential of these red algal extracts, and further studies are warranted to assess their efficacy in animal models.

## Data Availability

The original contributions presented in this study are included in the article/supplementary material. Further inquiries can be directed to the corresponding author.

## Supplementary Materials

The following supporting information can be downloaded at: https://www.mdpi.com/article/10.3390/tropicalmed11020041/s1, Figure S1: Effect of extracts solvent on Caco-2 and HEK-293T cell viability up to 48 and 72 h of incubation. Data were obtained by alamarBlue@ assay and are presented as mean ± SD from two independent experiments (*n* = 2) each carried out in quadruplicate. Figure S2: In vitro toxicity of *Osmundea pinnatifida* extract in (A) Caco-2 cells and (B) HEK-293T cells up to 72 h of incubation. Cell viability was determined using resazurin and is presented as % of control. The 50% cytotoxic concentration (CC_50_) was calculated by non-linear regression curve-fitting from two independent experiments. Figure S3: HEK-293T cells after incubation with *O. pinnatifida* extract at (A) 10 µg/mL, (B) 50 µg/mL, (C) 100 µg/mL, (D) 200 µg/mL, and (E) 0 µg/mL (cell control) for 72 h.

## Abbreviations

The following abbreviations are used in this manuscript:

CVA12	Coxsackievirus A12
LV	Lentivirus
HIV	Human immunodeficiency virus
MERS-CoV	Middle East respiratory syndrome coronaviruses
SARS-CoV-2	Severe acute respiratory syndrome 2
HSV	Herpes simplex virus
Caco-2	Human Colorectal Adenocarcinoma cells Caco-2
ATCC	American Type Culture Collection
DMEM	Dulbecco’s Modified Eagle’s Medium
FBS	Fetal bovine serum
eGFP	Green fluorescent protein
qPCR	Quantitative real-time polymerase chain reaction
DAPI	4′,6-Diamidino-2-phenylindole
CC_50_	50% cytotoxic concentration
EV71	Enterovirus 71
