# Supplementary material for: In Vitro Antiviral Activity of Red Algae Extracts from Chondracanthus teedei var. lusitanicus and Osmundea pinnatifida Against Coxsackievirus A12 and a Lentiviral Vector
Source: Trop Med Infect Dis. 2026 Jan 31;11(2):41. doi: 10.3390/tropicalmed11020041 (PMC12945184; doi:10.3390/tropicalmed11020041)
Supplement: Supplementary file 1 [file tropicalmed-11-00041-s001.zip › tropicalmed-4101578-supplementary.pdf]

Supplementary Materials

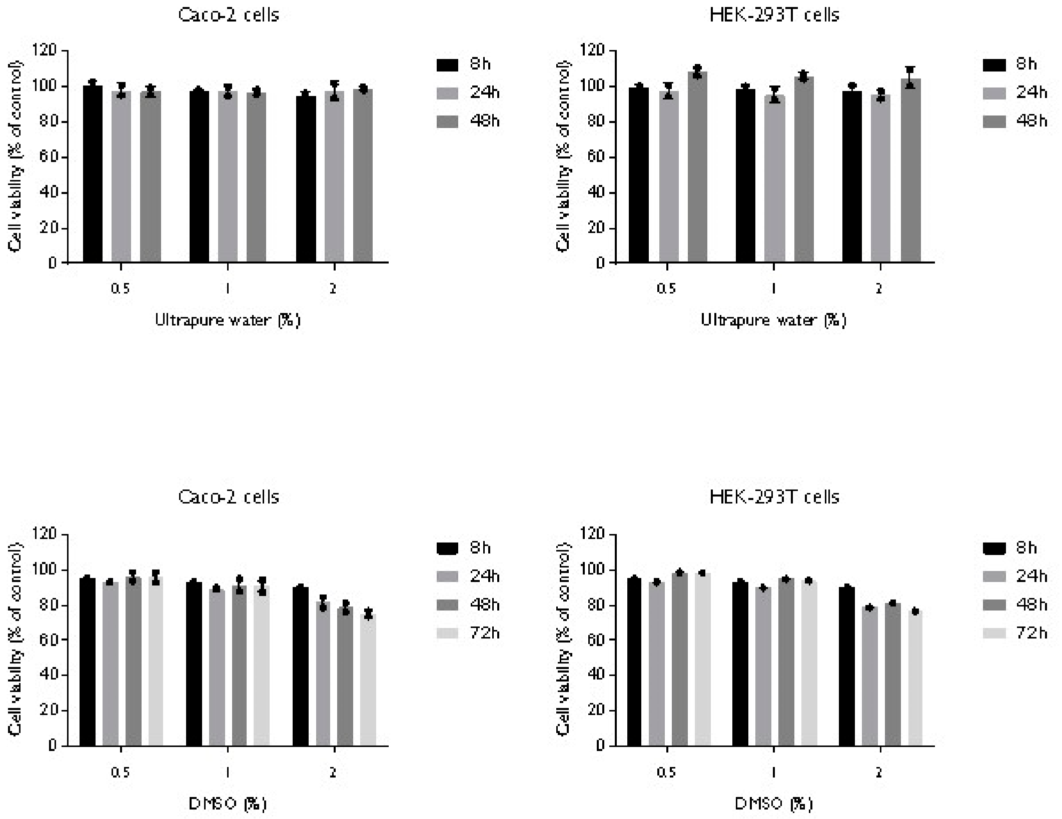

Figure S1. Effect of extracts solvent on Caco-2 and HEK-293T cell viability up to 48 and 72 h of incubation. Data were obtained by alamarBlue@ assay and are presented as mean + SD from two independent experiments (n = 2) each carried out in quadruplicate.

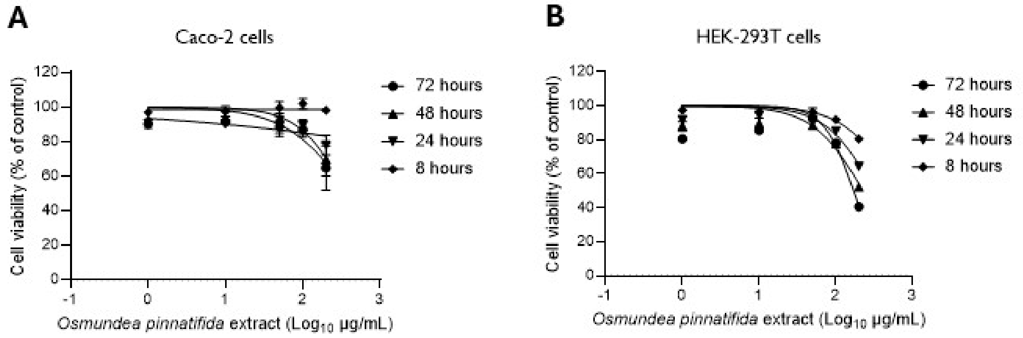

Figure S2. *In vitro t*oxicity of Osmundea pinnatifida extract in (**A**) Caco-2 cells and (**B**) HEK-293T cells up to 72 h of incubation. Cell viability was determined using resazurin and is presented as % of control. The 50% cytotoxic concentration (CC50) was calculated by non-linear regression curve-fitting from two independent experiments.

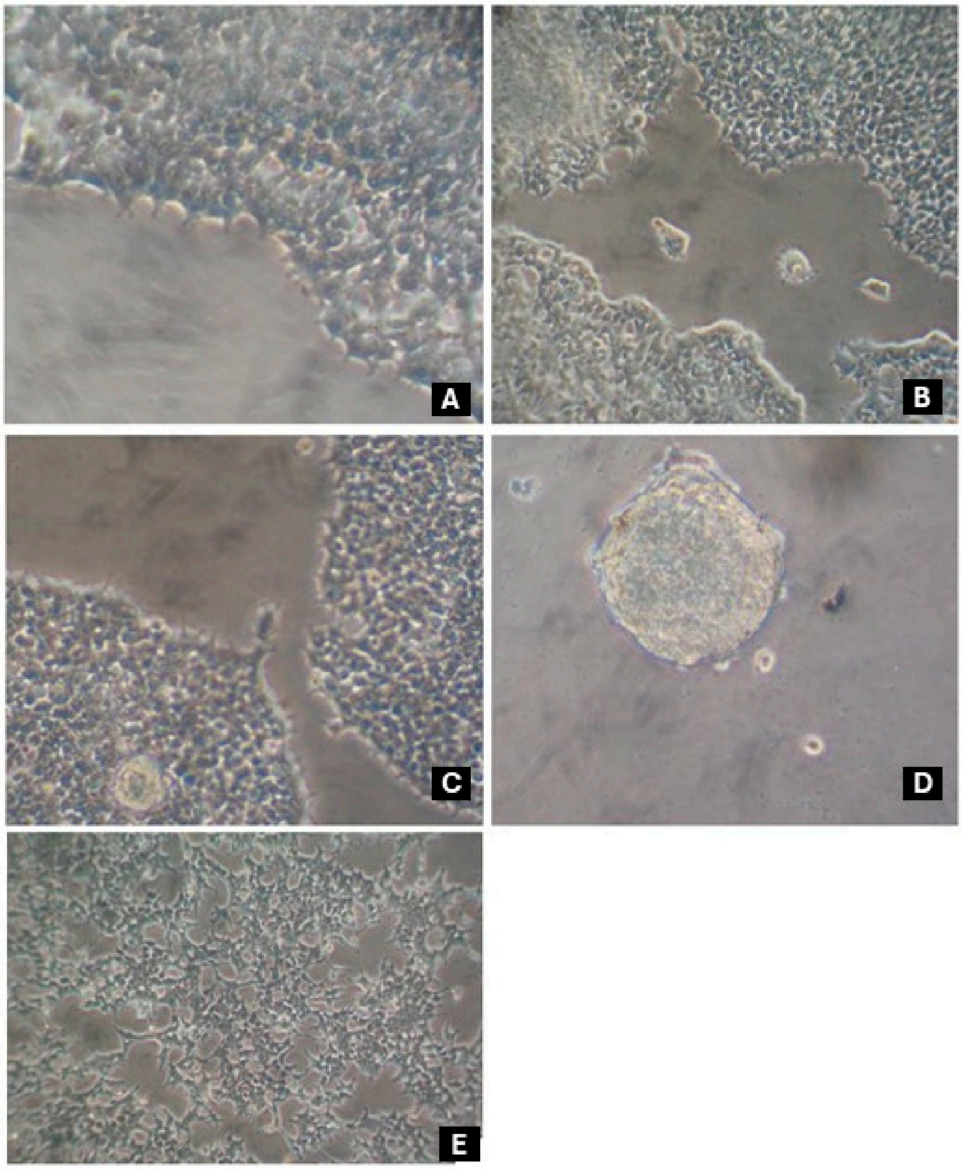

Figure S3. HEK-293T cells after incubation with O. pinnatifida extract at (**A**) 10 µg/mL, (**B**) 50 µg/mL, (**C**) 100 µg/mL, (**D**) 200 µg/mL, and (**E**) 0 µg/mL (cell control) for 72 h.
